# Characterizing severe obesity in children and youth referred for weight management

**DOI:** 10.1186/1471-2431-14-154

**Published:** 2014-06-19

**Authors:** Hebah A Salawi, Kathryn A Ambler, Rajdeep S Padwal, Diana R Mager, Catherine B Chan, Geoff D C Ball

**Affiliations:** 1 Department of Agricultural, Food and Nutritional Science, University of Alberta, Edmonton, AB T6G 2R3, CANADA; 2 Department of Pediatrics, University of Alberta, Edmonton, AB T5K0L4, CANADA; 3 Department of Medicine, University of Alberta, Edmonton, AB T6G 2R7, CANADA; 4 Department of Pediatrics, University of Alberta, Edmonton, AB T6G 2R3, CANADA; 5 Department of Physiology, University of Alberta, Edmonton, AB T6G 2R3, CANADA; 6 8B, Pediatric Centre for Weight and Health, Edmonton General Continuing Care Centre, 11111 Jasper Ave, Edmonton, AB T5K0L4, CANADA

**Keywords:** Severe obesity, Pediatric, Cardiometabolic risk, Nutrition, Physical activity, Canada

## Abstract

**Background:**

Severe obesity (SO) in pediatrics has become increasing prevalent in recent decades.

The objective of our study was to examine differences in demographic, anthropometric, cardiometabolic, and lifestyle variables in children and youth with SO *versus* their less overweight/obese (OW/OB) peers.

**Methods:**

A retrospective medical record review of 6-19 year old participants enrolled in an outpatient pediatric weight management clinic was conducted. SO (body mass index [BMI] ≥99^th^ percentile) and OW/OB (BMI ≥85^th^ and <99^th^ percentile) groups were created according to Centers for Disease Control and Prevention definitions. Demographic, anthropometric, cardiometabolic and lifestyle data reported at baseline (pre-intervention) were retrieved.

**Results:**

Of the 345 participants, most were girls (56.2%), Caucasian (78.7%), and had family incomes > $50,000/year (65.7%). The SO group (n = 161) had lower HDL-cholesterol and higher liver enzymes, insulin resistance and blood pressure than the OW/OB group (n = 184; all p < 0.01). They also reported higher total energy intakes, fewer steps/day, less moderate-to-vigorous physical activity, and more leisure time screen time (all p < 0.02) than their leaner peers. Compared to the OW/OB group, a higher proportion of individuals in the SO group possessed cardiometabolic risk factors, including high triglycerides (45.8% *vs* 58.5%), alanine aminotransferase (55.4% *vs* 81.4%), insulin resistance (55.6% *vs* 82.1%), systolic blood pressure (11.5% *vs* 27.3%), diastolic blood pressure (17.8% *vs* 30.0%), and low HDL-cholesterol (44.6% *vs* 64.6%; all p < 0.02). Aside from the ~75% of participants (groups combined) who met the daily recommended intakes of grain and meat products, <50% of boys and girls met any of the remaining nutrition and physical activity-related recommendations. Compared to the OW/OB group, greater proportions of children and youth in the SO group failed to meet moderate-to-vigorous physical activity (48.4% *vs* 31.9%) and leisure-time-screen-time recommendations (43.4% *vs* 28.3%; both p < 0.05).

**Conclusion:**

Children and youth with SO have a worse cardiometabolic profile and less favorable lifestyle habits than their OW/OB peers. These differences emphasize the heightened obesity-related health risks associated with SO in the pediatric years.

## Background

Obesity is one of the most common chronic disorders affecting the health and well-being of children and youth and its prevalence remains high [1,2]. Recent data from the Canadian Health Measures Survey revealed that almost one-third of Canadian 5 – 17 year-olds are either overweight or obese [3]. Even more striking is recent evidence from the US indicating an increasing prevalence of severe obesity (SO). For example, between the National Health and Nutrition Examination Survey (NHANES) 1988-1994 and NHANES 1999-2004, the prevalence of SO in children and youth increased by 72% from 2.2% to 3.8% [4]. With evidence suggesting that the overall prevalence of pediatric overweight and obesity has remained stable [2], these data are cause for concern because SO is associated with even worse cardiometabolic risk factors profiles, including elevated total cholesterol, triglycerides, serum glucose, and systolic blood pressure and low HDL-cholesterol [2,5,6]. Compounding these health risks is the observation that SO tends to be more challenging for clinicians and families to manage successfully. For instance, a lower degree of obesity predicts successful weight loss during pediatric weight management [7,8], suggesting that as the degree of obesity in children and youth increases, it becomes more entrenched and resistant to therapeutic interventions.

Recent reports have highlighted a growing research and clinical focus on SO in pediatrics, with data characterizing high prevalence levels [2,4-9] and cardiometabolic health risks [10-14]. This information is highly relevant from a health services perspective because many children and youth referred to weight management clinics present with SO and because most clinical programs use the severity of obesity and/or presence of obesity-related co-morbidities as referral criteria [11]. Clinical practice guidelines [12] and expert recommendations [13] highlight the importance of making positive lifestyle (nutrition and physical activity) changes in order to manage obesity. With this in mind, the objectives of our research were to characterize and examine differences in demographic, anthropometric, cardiometabolic and lifestyle variables in children and youth with SO *versus* their less overweight and obese (OW/OB) peers among a cohort of individuals referred for weight management.

## Methods

### Participants, setting, and procedures

A cross-sectional study design was used to assess demographic, anthropometric, cardiometabolic and lifestyle variables of children and youth. We included 6- to 19-year-olds with an age- and sex-specific BMI ≥85^th^ percentile [15]. Data from individuals were excluded if they were *(i)* outside of our defined age range or *(ii)* not ambulatory (*i.e.,* in a wheelchair). Only data collected before weight management was initiated were included. Participants were ambulatory and otherwise healthy individuals who were referred by physicians to an outpatient, multidisciplinary weight management clinic (Pediatric Centre for Weight and Health, Stollery Children’s Hospital, (PCWH), Edmonton, AB) between April 2005 and December 2011. Family-centered interventions offered at the PCWH take a lifestyle and behavioral focus and include both individual and group-based programming, the details of which have been reported [16-18]. All data were retrieved retrospectively via medical record review, which included a systematic protocol for data capture, entry, management, and audit [14]. Site approval for this research was received by Alberta Health Services and research ethics approval was granted by the Health Research Ethics Board at the University of Alberta (Edmonton, AB).

### Anthropometry and demography

Wearing only light clothing and without shoes, weight was measured to the nearest 0.1 kg with a medical digital balance scale (SECA 644, Hanover, MD) and height was measured to the nearest 0.1 cm using a digital stadiometer (SECA 242 stadiometer, Hanover, MD). Subsequently, weight and height data were entered into *EpiInfo™* (version 5.3.1, 2008; Centers for Disease Control and Prevention; Atlanta, GA) to calculate body mass index (BMI), BMI percentile, and BMI z-score. Waist circumference was measured to the nearest 0.1 cm at the iliac crest using a spring-loaded Gulick anthropometric tape (FitSystems; Calgary, AB). A standardized questionnaire was used to collect demographic data from parents who provided information regarding their family income as well as their sons’ and daughters’ dates of birth, gender, and ethnicity.

### Cardiometabolic measurements

Although samples were taken at several clinic locations in the Edmonton-area, all analyses were completed at the University of Alberta Hospital outpatient laboratory. Glucose was analyzed with a Beckman LX20 analyzer; all other variables were measured with electrochemiluminescence (Elecsys 2010, Roche, Basel, Switzerland). After a 10 – 12 hour fast, participants provided a blood sample in order to determine a panel of cardiometabolic risk factors that included total cholesterol, HDL-cholesterol, LDL-cholesterol, triglycerides, alanine aminotransferase (ALT), glucose, and insulin. The total cholesterol/HDL-cholesterol ratio was calculated. Similarly, the homeostatic model assessment of insulin resistance (HOMA-IR) was used to derive a measure of insulin resistance (IR) according to the following formula: fasting insulin (mU/L) × fasting glucose (mM)/22.5 [19]. Systolic and diastolic blood pressures (SBP and DBP, respectively) were measured manually while participants were seated, after a 5-minute rest, by a clinician using a sphygmomanometer and an appropriately-sized arm cuff, all of which were in accordance with Canadian guidelines [20]. Five minutes later, another measure was taken; when SBP differed ≥10 mm Hg between the two measurements, the lower value of the two was recorded.

### Nutrition and physical activity assessments

Prospective measurements of dietary intake were made using 4-day food records, which included 1-2 weekend days. Families completed the records, which were reviewed by the clinic dietitian in order to reduce the likelihood of food and beverage omissions and to verify brand names and portion sizes. The *Food Processor Diet Analysis Software SQL* (version 10.0.0, ESHA Research; Salem, OR) program was used to analyze the data and calculate average daily intakes. The software was not able to provide food group data in accordance with *Canada’s Food Guide*[21], so the clinic dietitian manually calculated the number of daily servings of Vegetables and Fruit, Grain Products, Milk and Alternatives, and Meat and Alternatives.

The New Lifestyles Digi-Walker SW-200 pedometer (Lee’s Summit, MO) was used to assess physical activity over a 7-day period including 1-2 weekend days. During the first visit, the PCWH exercise specialist instructed participants and parents on correct pedometer placement, calibration, and data-monitoring procedures. In advance, all pedometers underwent a 100 step accuracy test. Only those units that recorded ≥99% accuracy were used for physical activity assessment. On the second visit, pedometer log books were reviewed with families for accuracy and completeness. Since pedometers provide an overall estimate of physical activity that does not include intensity, the 7-day physical activity recall interview [22] was used to assess the amount of time participants spent engaged in moderate -to- vigorous physical activity (MVPA); MVPA represents higher- intensity physical activities (*e.g.,* brisk, walking, skating, bike riding, running, basketball, soccer), which may be particularly important for pediatric weight management [23,24]. Accordingly, *moderate*, *hard*, and *very hard physical activity* were calculated for each participant and summed to derive MVPA. The exercise specialist conducted all interviews, during which information regarding screen time (including leisure time television viewing, movie rental and (or) theatre viewing, video game playing, and computer time) as a proxy measure of sedentary activity and sleep duration over the previous seven days was also collected. Children and parents were interviewed together, whereas adolescents completed the interviews independently. The exercise specialist reviewed all records with families after they were completed to verify responses and ensure the accuracy of the information they contained.

### Weight status, cardiometabolic risk factor, and lifestyle habit definitions

Overweight/obesity (OW/OB) was defined as an age- and sex-specific BMI ≥85^th^ to <99^th^ percentile whereas SO was defined as an age- and sex-specific BMI ≥99^th^ percentile [15]. A number of recent reports have used the same criteria to define SO in children and adolescents [4,25-27]. Criteria for dichotomizing cardiometabolic risk factors and lifestyle habits to determine relatively high/low health risk are summarized in Table 1.

**Table 1:** Cardiometabolic risk factor cut-offs and lifestyle recommendations

**Variable**	**Cut-off**	**Variable**	**Recommendation**
WC	≥90^th^ percentile [28]	V & F	5-8 servings/day [29]
TC	≥5.2 mmol/L [30]	Grains	4-7 servings/day [29]
TG	≥1.24 mmol/L [28]	Milk	2-4 servings/day [29]
HDL-C	≤1.03 mmol/L [28]	Meat	1-3 servings/day [29]
LDL-C	≥3.4 mmol/L [31]	Steps	Children [32]:
			Boys: 13,000-15,000 steps/d
			Girls: 11,000-12,000 steps/d
			Adolescents [30]:
			10,000-11,700 steps/d
ALT	<20 U/L [33]	MVPA	≥60 minutes/day [34]
HOMA-IR	≥3.16 mmol/L [19]	LTST	≤2 hours/day [35]
SBP	≥90^th^ percentile [36]	Sleep	Children: ≥10 hr/night [37]
DBP	≥90^th^ percentile [36]		Adolescents: ≥9 hr/night [37]

WC (waist circumference), TC (total cholesterol), TG (triglycerides), HDL-C (high density lipoprotein cholesterol), LDL-C (low density lipoprotein cholesterol), ALT (alanine aminotransferase), HOMA-IR (homeostatic model assessment of insulin resistance), SBP (systolic blood pressure), DBP (diastolic blood pressure), V & F (Vegetables & Fruit), Grains (Grain Products), Milk (Milk & Alternatives), Meat (Meat & Alternatives), Steps, MVPA (moderate-to-vigorous physical activity), LTST (leisure time screen time), Sleep (sleep duration).

### Statistical analysis

Continuous variables were reported using means and standard deviations whereas categorical data were reported as proportions. Group comparisons of continuous variables were conducted using independent samples t-tests (OW/OB vs SO groups). The Chi-square test was used to compare the proportions of participants (OW/OB *vs* SO groups) *(i)* across high/low cardiometabolic risk factors and *(ii)* between individuals who met/did not meet nutrition and physical activity-related recommendations. For descriptive purposes, also we completed stratified analyses using multivariable analysis of variance across all *(i)* cardiometabolic risk factors and *(ii)* nutrition and physical activity-related variables to examine weight status (OW/OB vs SO groups) by *(i)* gender (boys *vs* girls), *(ii)* age group (children [<12 years old] *vs* youth [12 years old]), and *(iii)* ethnicity (Caucasian *vs* non-Caucasian) interaction effects. Group differences were considered statistically significant at a p-value <0.05 and all data were analyzed using *SPSS* (version 19; IBM SPSS Statistics).

## Results

In total, 345 participants were included in this report, the majority of whom were girls (56.2%), Caucasian (78.7%), and had family incomes > $50,000 CDN/year (65.7%). The anthropometric characteristics of children and youth grouped according to OW/OB and SO categories are presented in Table 2. By design, the SO group was both heavier and more overweight than their OW/OB peers. We also completed exploratory analyses between OW (n = 26) and OB (n = 158) sub-groups. Among all of the demographic, cardiometabolic, and lifestyle variables, the only group differences related to systolic blood pressure (OW: 119 ± 13mmHg *versus* OB: 110 ± 11 mmHg; p < 0.05) and dietary energy from saturated fat (OW: 203 ± 78 kcal/d *versus* OB: 254 ± 119 kcal/d; p < 0.05). These findings highlighted the homogeneity within the OW/OB group and justified our decision to combine these sub-groups.

**Table 2:** Anthropometric characteristics of children and youth categorized as overweight/obese (OW/OB) and severely obese (SO)

	**OW/OB**	**SO**	**p-value**
Age (y)	12.8 ± 2.8	12.3 ± 2.9	0.07
	(n = 184)	(n = 161)	
Height (cm)	155.9 ± 13.0	157.9 ± 14.5	0.2
	(n = 184)	(n = 161)	
Weight (kg)	71.2 ± 19.8	94.4 ± 29.3	<0.001
	(n = 184)	(n = 161)	
BMI (kg/m^2^)	28.7 ± 4.1	36.9 ± 6.7	<0.001
	(n = 184)	(n = 161)	
BMI Percentile	97.1 ± 2.3	99.5 ± 0.2	<0.001
	(n = 184)	(n = 161)	
BMI Z-Score	2.0 ± 0.3	2.6 ± 0.2	<0.001
	(n = 184)	(n = 161)	
Waist Circumference (cm)	88.0 ± 11.0	104.6 ± 15.6	<0.001
	(n = 184)	(n = 153)	

Data are presented as mean ± standard deviation. BMI (body mass index).

Note: Sample sizes vary variable-to-variable because of an incomplete dataset.

**Figure 1:**
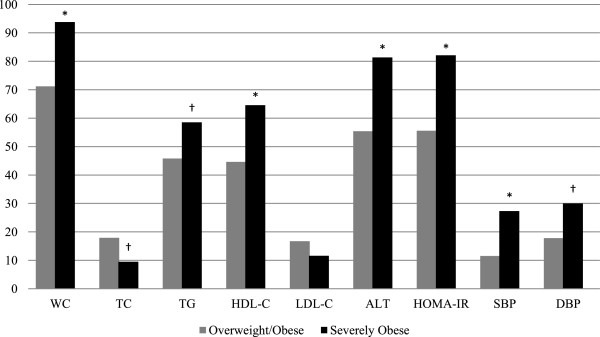
**The prevalence of abnormal cardiometabolic risk factors in overweight/obese (OW/OB) and severely obese (SO) groups.** WC (waist circumference), TC (total cholesterol), TG (triglycerides), HDL-C (high density lipoprotein cholesterol), LDL-C (low density lipoprotein cholesterol), ALT (alanine aminotransferase), HOMA-IR (homeostatic model assessment of insulin resistance), SBP (systolic blood pressure), DBP (diastolic blood pressure). *p ≤ 0.001. † p ≤ 0.05.

Comparisons of cardiometabolic risk factors (continuous data) between OW/OB and SO groups are shown in Table 3, with lower TC and HDL-C as well as higher ALT, fasting insulin, HOMA-IR, SBP, and DBP in the SO *vs* OW/OB group. After we dichotomized cardiometabolic risk factors (see Table 1 for thresholds), we found that the proportions of children and youth with cardiometabolic risk factors were higher in the SO group compared to the OW/OB group for most variables, including WC, TG, HDL-C, ALT, HOMA-IR, SBP and DBP (see Figure 1).

**Table 3:** Cardiometabolic risk factors of children and youth categorized as overweight/obese (OW/OB) and severely obese (SO)

	**OW/OB**	**SO**	**p-value**
Triglycerides (mmol/L)	1.4 ± 0.8	1.5 ± 0.6	0.2
	(n = 168)	(n = 147)	
Total cholesterol (mmol/L)	4.5 ± 0.9	4.3 ± 0.8	0.03
	(n = 168)	(n = 147)	
HDL-cholesterol (mmol/L)	1.1 ± 0.3	1.0 ± 0.2	<0.001
	(n = 168)	(n = 147)	
LDL-cholesterol (mmol/L)	2.7 ± 0.8	2.6 ± 0.7	0.1
	(n = 168)	(n = 147)	
Total cholesterol/HDL ratio	4.3 ± 1.2	4.5 ± 1.1	0.2
	(n = 168)	(n = 147)	
ALT (U/L)	24.9 ± 16.0	31.6 ± 17.8	0.001
	(n = 157)	(n = 145)	
Fasting glucose (mmol/L)	4.9 ± 0.4	4.9 ± 0.4	0.7
	(n = 159)	(n = 138)	
Fasting insulin (mU/L)	18.5 ± 15.8	31.3 ± 20.4	0.02
	(n = 154)	(n = 130)	
HOMA-IR	4.0 ± 3.4	6.8 ± 4.6	0.03
	(n = 151)	(n = 123)	
Systolic BP (mmHg)	108 ± 9	115 ± 11	0.01
	(n = 174)	(n = 150)	
Diastolic BP (mmHg)	69 ± 8	72 ± 9	0.01
	(n = 174)	(n = 150)	

Data are presented as mean ± standard deviation. HDL-C (high density lipoprotein cholesterol), LDL-C (low density lipoprotein cholesterol), ALT (alanine aminotransferase), HOMA-IR (homeostatic model assessment of insulin resistance).

Note 1: Sample sizes vary variable-to-variable because dataset was incomplete.

Note 2: Group comparisons for fasting glucose, fasting insulin, HOMA-IR, systolic BP and diastolic BP were completed using non-parametric analyses since these variables were not normally distributed.

Comparisons of nutrition- and physical activity-related behaviors between the SO and OW/OB groups are shown in Table 4. Overall, the SO group consumed a greater amount of total energy, which was reflected in their higher intakes of all macronutrients (carbohydrate, protein, and fat) as well as servings/day of Grain Products and Meat and Alternatives. In addition, in relation to their OW/OB peers, the SO group reported accumulating fewer daily steps, less MVPA, and more LTST.

**Table 4:** Nutrition- and physical activity-related habits of children and youth categorized as overweight/obese (OW/OB) and severely obese (SO)

	**OW/OB**	**SO**	**p-value**
Energy intake (kcal/d)	1955 ± 551	2333 ± 857	<0.001
	(n = 166)	(n = 139)	
Carbohydrate intake (g/d) ‡	261 ± 83	294 ± 110	0.004
	(n = 166)	(n = 139)	
Protein intake (g/d) ‡	77 ± 32	93 ± 43	<0.001
	(n = 166)	(n = 139)	
Fat intake (g/d) ‡	68 ± 25	89 ± 42	<0.001
	(n = 166)	(n = 139)	
Vegetables and Fruit (servings/d)	3.6 ± 1.9	3.6 ± 2.4	0.8
	(n = 156)	(n = 132)	
Grain Products (servings/d)	7.7 ± 2.6	8.8 ± 4.0	0.01
	(n = 156)	(n = 132)	
Milk and Alternatives (servings/d)	2.0 ± 1.0	2.2 ± 1.3	0.4
	(n = 156)	(n = 132)	
Meat and Alternatives (servings/d)	1.9 ± 0.8	2.6 ± 1.4	0.01
	(n = 155)	(n = 132)	
Steps/d	8012 ± 3456	7049 ± 3127	0.02
	(n = 154)	(n = 120)	
MVPA (min/d)	69.1 ± 52.8	50.5 ± 41.8	0.007
	(n = 159)	(n = 119)	
Active video game time (min/d)	2.9 ± 14.1	1.8 ± 7.8	0.3
	(n = 158)	(n = 117)	
Leisure time screen time (min/d)	163.5 ± 117.8	217.6 ± 131.2	0.04
	(n = 159)	(n = 120)	
Sleep duration (hr/night)	9.6 ± 1.0	9.5 ± 1.0	0.4
	(n = 163)	(n = 125)	

Data are presented as mean ± standard deviation. MVPA (moderate-to-vigorous physical activity).

Note 1: Sample sizes vary variable-to-variable because dataset was incomplete.

Note 2: Group comparisons for Vegetables and Fruit, Grain Products, Milk and Alternatives, Meat and Alternatives, MVPA, Active video game time, and Leisure time screen time were completed using non-parametric analyses since these variables were not normally distributed.

‡Differences in macronutrients (carbohydrate, protein, and fat) remained significant after adjustment for total energy intake.

**Figure 2:**
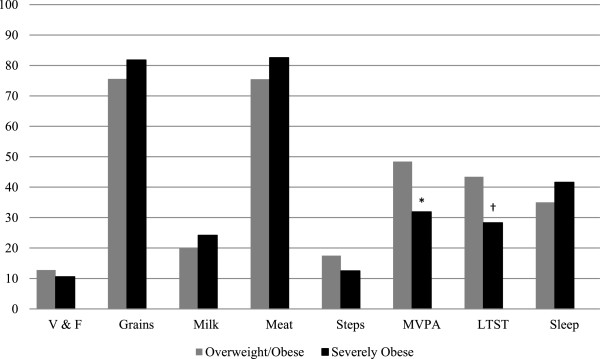
**The proportion of participants in overweight/obese (OW/OB) and severely obese (SO) groups that achieved recommendations for nutrition and physical activity behaviours.** V & F (Vegetables and Fruit), MVPA (moderate-to-vigorous physical activity), LTST (leisure time screen time). *p ≤ 0.01. † p ≤ 0.05.

The proportions of participants in the OW/OB and SO groups that met each of the nutrition- and physical activity-related recommendations (defined in Table 1) are shown in Figure 2. While we failed to observe any significant group differences according to the food groups, in general, a large proportion (70 – 80%) of our sample met the recommendations for daily intakes of Grain Products and Meat and Alternatives whereas a small proportion (10 – 25%) satisfied the Vegetables and Fruit and Milk and Alternatives guidelines. Similar to the nutrition-related recommendations, we observed that most children and youth in our study did not satisfy the physical activity-related recommendations. However, almost twice as many OW/OB participants met the MVPA recommendation compared to their SO peers (χ^2^ = 7.6; p < 0.01). Similar differences were revealed with a greater proportion of the OW/OB group meeting LTST recommendations (χ^2^ = 6.7; p < 0.05) than the SO group.

Our stratified analyses revealed no significant *(i)* weight status (OW/OB *vs* SO) × gender (boys *vs* girls), *(ii)* weight status × age group (children *vs* youth), or *(iii)* weight status × ethnicity (Caucasian *vs* non-Caucasian) interactions across our cardiometabolic risk factors and nutrition and physical activity-related habits (all interaction effects >0.05).

## Discussion

In our study of children and youth referred for weight management, we showed that *(i)* those with SO were at increased cardiometabolic health risk compared to their OW/OB peers, and *(ii)* while the study sample as a whole had sub-optimal nutrition and physical activity habits, those with SO tended to have poorer lifestyle behaviors than their OW/OB counterparts.

Our observations are consistent with those of Skelton *et al.*[4] who applied the same classification system to define weight status categories in a sample (n ~ 12,000) of boys and girls from the National Health and Nutrition Examination Surveys II, III, and 1999-2004 and also reported a higher degree of cardiometabolic risk among the SO group in relation to their OW/OB peers. To our knowledge, we are the first to report differences in lifestyle behaviors between children and youth with SO *versus* their OW/OB peers. This is a noteworthy finding and emphasizes the point that a one-size-fits-all approach to weight management is inappropriate since those with SO appear to have greater potential to improve their lifestyle habits, which may have led to their higher degree of obesity. Viewed another way, the less favorable lifestyle habits of the SO group provides an opportunity for them to make positive improvements to nutrition and physical activity that can contribute to weight management success and improvements in other health outcomes, independent of substantial weight loss. For instance, a recent review of 22 studies of immersion programs examined treatments that combined a controlled diet, physical activity, nutrition education and therapy and/or education supportive of behavioral change. The participants in the reviewed treatments lost an average of 23.9% of their overweight during treatment and 20.6% during follow up [38]. Although there are exceptions [39], we recently reported [16] that it is challenging for most boys and girls with obesity to improve their weight status by making modest lifestyle changes; for many individuals, preventing further weight gain by making nutrition and physical activity changes that lead to weight stabilization is an important and achievable goal, albeit one that may or may not satisfy the expectations and desires of families [40].

Despite the differences we observed between SO and OW/OB groups, overall, most children and youth in our study did not achieve current lifestyle recommendations. In addition to supporting previous findings from our clinic [41], these results are aligned with other larger, population-based studies. For instance, Shields showed low levels of vegetable and fruit intakes as a reflection of poor diet quality that may increase the risk of pediatric obesity [42]. Similarly, Garriguet and colleagues found that a substantial number of children and youth do not meet the recommendations for vegetables and fruit, and milk and alternatives intake [21,43]. He *et al.* suggested that increased consumption of fruits and vegetables might reduce long-term weight gain and obesity risk [44]. Lowering the amount of added fructose in diets may also benefit children and youth with obesity by improving their markers for cardiometabolic dysfunction, which may be particularly beneficial for those at risk for developing type 2 diabetes or nonalcoholic fatty liver dysfunction [45]. Although our data are cross-sectional, our observations suggest that interventions that emphasize improvements in diet quality and quantity are likely to be important strategies for managing pediatric obesity [12].

It is clear that a physically active lifestyle has numerous health promoting benefits (beyond weight management) [46], but it remains a challenge for most boys and girls to achieve current recommendations. *The Canadian Physical Activity and Sedentary Behaviour Guidelines*[34] suggest that children and youth should accumulate ≥60 minutes of daily MVPA and limit their sedentary time to <2 hours daily [47]. Following these guidelines can help improve body composition, cardiorespiratory and musculoskeletal health, social behaviors, self-esteem and academic success [48]. A relatively high proportion (30 – 40% according to the self-reported data) of our sample met the physical activity recommendations; however, based on objective physical activity data collected using accelerometers, <10% of Canadian children and youth accumulate ≥60 minutes of daily MVPA [49]. The difference in the proportion of individuals meeting MVPA recommendations in our sample *versus* national normative data suggests boys and girls in our study may have overestimated their physical activity levels. Our observation that a lower percentage of our sample achieved the daily steps recommendation *versus* self-reported MVPA provides indirect evidence in support of this point, but is validated by other data showing that young individuals may overestimate their physical activity using self-report tools [50].

Our study has several limitations to acknowledge. First, since all of the participants were referred by physicians to a weight management clinic, our findings may not apply to all children and youth with SO, especially those who do not enroll in health services for obesity management. Notably, our clinic is one of eight that is participating in a new Canadian multi-center study to characterize children, youth and families receiving health services for managing pediatric obesity [51]. Evidence generated from this registry of ~1,600 participants will show whether our findings are generalizable to other similar settings. Second, given our cross-sectional study design, we cannot comment on whether our observed differences between SO and OW/OB groups remain consistent over time or whether changes in response to therapy are similar across groups. Third, while our analyses included objective measurements of anthropometric variables and cardiometabolic risk factors, self-reported nutrition and physical activity data are subjective in nature, which can reduce measurement reliability and accuracy. The fact that we still observed group differences based on these self-reported data provides some confidence that real differences exist between SO and OW/OB groups. Finally, a recent report [52] highlighted the need for consistent terms and definitions of SO in pediatrics. While the current manuscript applied one of several different definitions of SO in the literature, applying the newer recommended criteria in future studies is needed to establish its superiority.

## Conclusions

In conclusion, our study revealed that children and youth with SO were at increased cardiometabolic health risk and tended to have less healthy nutrition and physical activity habits compared to their OW/OB counterparts. In their discussions with families, clinicians can use this information to highlight the health risks associated with a higher degree of obesity. By applying family- and client-centered counseling strategies [53,54], they can also emphasize the potential that exists for boys and girls with obesity to make healthy changes since most do not satisfy current lifestyle recommendations.

## Competing interest

The authors declare that they have no competing interest.

## Authors’ contributions

HS analyzed the data and co-authored the first draft of the manuscript with GDCB; KAA assisted with data collection and management; RSP, DRM, and CC provided critical feedback and edits on data analysis, data interpretation, and manuscript presentation; GDCB conceived the study, assisted with data analysis and interpretation, and co-authored the first draft of the manuscript with HS. All authors contributed to writing the manuscript. All authors reviewed and approved the final draft of the manuscript prior to submission.

## Pre-publication history

The pre-publication history for this paper can be accessed here:

http://www.biomedcentral.com/1471-2431/14/154/prepub
